# Acute Abdomen as a Clinical Presentation of COVID-19-Associated Multisystem Inflammatory Syndrome in Children

**DOI:** 10.1155/2024/9941131

**Published:** 2024-07-31

**Authors:** Elmira Hajiesmaeil Memar, Fatemeh Tahghighi, Sedigheh Yousefzadegan, Parisa Sadeghirad, Ashraf Mousavi, Ramin Zare Mahmoudabadi, Hossein Saeidi, Mehri Ayati, Sahar Naderi, Sara Memarian, Seyedmusa Zeinalabedin, Bahar Ashjaei, Hojatollah Raji, Leila Tahernia, Hosein Alimadadi, Vahid Ziaee

**Affiliations:** ^1^ Department of Pediatrics Tehran University of Medical Sciences, Tehran, Iran; ^2^ Children's Medical Center Pediatric Center of Excellence, Tehran, Iran; ^3^ Department of Pediatric Iran University of Medical Sciences, Tehran, Iran; ^4^ Firoozabadi Hospital Iran University of Medical Sciences, Tehran, Iran; ^5^ Firoozabadi Clinical Research and Development Unit (FACRDU) Iran University of Medical Sciences, Tehran, Iran; ^6^ Department of Pediatrics Semnan University of Medical Sciences, Semnan, Iran; ^7^ Department of Surgery Tehran University of Medical Sciences, Tehran, Iran; ^8^ Pediatric Rheumatology research group Rheumatology Research Center Tehran University of Medical Science, Tehran, Iran; ^9^ Pediatric Rheumatology Society of Iran, Tehran, Iran

## Abstract

**Background:**

On December 2019, a novel coronavirus disease (COVID-19) spread worldwide and became a pandemic. Multisystem inflammatory syndrome in children (MIS-C) due to cytokine release syndrome following COVID-19 presents with various manifestations. We hypothesize that one of the rare manifestations is acute abdomen. *Case Presentation*. In this case series, eight cases (five girls and three boys) of gastrointestinal (GI) involvement and acute abdomen were reported to be associated with the cytokine storm due to COVID-19 infection. All patients were of Iranian nationality (Caucasian ethnicity), with a mean age of 8.9 years (range 3.5–14). They all presented with fever and acute abdominal pain. Additionally, maculopapular rash and edema of the extremities were common presentations. Free fluid on abdominal ultrasound or computerized tomography (CT) scan was observed in all patients. All cases tested positive for COVID-19. In six cases, laparotomy or abdominal surgery was performed for a diagnosis of acute abdomen, but appendicitis was confirmed in only one case. None of the cases presented with phlegmon. Elevated serum lipase and amylase levels were noted in two cases. Seven patients received corticosteroid pulse therapy. Clinical symptoms improved after one or two doses, and all patients were discharged after 4 weeks of follow-up with no mortality or morbidity.

**Conclusion:**

Patients experiencing unexplained acute abdominal pain along with fever, skin rash, and peripheral edema, who had a history of COVID-19 infection prior to or during the episode of acute abdomen symptoms, should be considered to have MIS-C. Furthermore, methylprednisolone pulse therapy could be a safe treatment option, reducing hospitalization duration in this patient population.

## 1. Introduction

On December 2019, a novel coronavirus disease (COVID-19) emerged in Wuhan, China, and rapidly spread globally. The World Health Organization (WHO) officially declared the outbreak a pandemic on March 2020 [1, 2]. COVID-19 is caused by severe acute respiratory syndrome coronavirus 2 (SARS-CoV-2), first identified in 2019. While the typical clinical features include mild to moderate symptoms such as fever and cough, some patients may experience severe respiratory manifestations [2]. Although pulmonary involvement is a prominent aspect of COVID-19, the disease can also present with rare and unique extrapulmonary manifestations, particularly in children.

COVID-19 infections in children are often asymptomatic; however, the potential complications can be severe and even fatal if not promptly recognized. Following a COVID-19 infection, there is a robust inflammatory response characterized by the overproduction of proinflammatory cytokines, known as a “cytokine storm” [3, 4]. Multisystem inflammatory syndrome in children (MIS-C) is a hyperinflammatory condition involving two or more organ systems and can manifest as a syndrome resembling Kawasaki disease [5, 6, 7, 8]. Children with hyperinflammatory status may exhibit fever, rash, conjunctivitis, edema, extremity pain, and gastrointestinal (GI) symptoms, sometimes progressing to pleural, pericardial, or ascitic effusions [7]. Cytokine storm and MIS-C are major causes of acute respiratory distress syndrome (ARDS) and multiple organ failure in COVID-19 patients [9]. The varying levels of cytokines and chemokines play a crucial role in the disease progression from mild to severe stages [8]. Viral replication results in tissue damage and excessive recruitment of innate and adaptive immune cells, which mediate a dysregulated hyperinflammatory response contributing to cytokine storm syndrome and organ damage [10]. This excessive inflammatory response results in a severe disease course and worsens prognosis, and it can be life-threatening in children. It is caused by the excessive and complex response of the immune system to an external stimulus [2, 9].

The gastrointestinal and hepatobiliary system is frequently affected in children experiencing cytokine storms, leading to presentations such as acute abdomen or other GI manifestations [11, 12]. This case series aims to highlight acute abdomen and clinical features of appendicitis as an atypical presentation of MIS-C.

## 2. Methods

All the cases presented in this case series have been referred to educational hospitals affiliated to the Tehran University of Medical Sciences, located in Tehran, Iran.

Literature review was done by extensive search in search engines such as PubMed, Google Scholar, and Embase. English language and keywords such as pediatrics, MIS-c, acute abdomen, and their combinations were used for the search. All of found articles were evaluated in terms of title, abstract, and full text, and after removing duplicate and irrelevant items, 24 articles related to the research were selected and reviewed.

In order to determine the infection, SARS-CoV-2 reverse transcription polymerase chain reaction (RT-PCR) was performed using the Premix Ex Taq™ (Probe qPCR, TaKaRa, Japan) following the manufacturer's instructions. The RT-PCR was performed according to the Centers for Disease Control and Prevention (CDC) protocol using the same primers and probes as in the CDC 2019-Novel Coronavirus (2019-nCoV) RT-PCR Diagnostic Panel. All tests were performed in one of the tertiary and referral medical centers in Tehran.

## 3. Cases Presentation

In this case series, eight pediatric cases (five girls and three boys) of the acute abdomen associated with complications of COVID-19 are addressed, and we believe their acute abdominal pain is due to COVID-19-associated MIS-c. All patients had Caucasian ethnicity (Iranian nationality), and the mean age was 8.9 years (range 3.5–14). First case was the patient that brought the issue to our attention. Most patients (87.5%) were older than 6 years, and just one patient was less than 5 years old. The symptoms and laboratory findings of all cases have been summarized in Table 1. The frequencies of clinical and paraclinical findings are shown in Table 2.

### 3.1. Case One

A 10-year-old girl presented with fever, conjunctivitis, and periorbital edema 4 days prior to admission, along with a history of a positive COVID-19 diagnosis 4 weeks earlier. She exhibited a maculopapular rash throughout her body, dry lips, strawberry tongue, abdominal pain, nausea, and vomiting. Physical examination revealed generalized abdominal tenderness, guarding, and tachypnea. Abdominal ultrasound indicated severe free fluid and bowel wall thickening. Due to suspected acute abdomen, a laparotomy was performed, revealing bowel wall thickening in the terminal ileum, short-term inflammation of the appendix tips, and serous fluid departure with abdominal lymphadenopathy on histopathology investigation. Cardiac echo results were normal. The patient was managed with intravenous (IV) fluid therapy and corticosteroid pulse therapy (30 mg/kg for 3 days) for MIS-C. Follow-up after 4 weeks showed resolution of symptoms, with normal echocardiography and laboratory findings except for mild thrombocytopenia (platelet count between 130,000 and 150,000 per microliter) at 1, 2, and 6 months.

### 3.2. Case Two

Case two involved a previously healthy 6-year-old boy who was admitted with 2 days of fever, abdominal pain, nausea, and vomiting. He had no recent history of respiratory illness or exposure to COVID-19 patients. Lumbar puncture (LP) was performed to rule out meningitis, and treatment with ceftriaxone and vancomycin was initiated. Both LP and COVID-19 PCR test results were negative. On the fifth day of illness, he continued to have fever, abdominal pain, bilateral conjunctivitis, and edema in his hands and feet. Echocardiography revealed a right coronary artery (RCA) aneurysm. Intravenous immunoglobulin (IVIG) therapy (1 g/kg/day) was initiated due to suspicion of Kawasaki disease.

By the sixth day of illness, the patient was afebrile but experienced generalized abdominal pain with tenderness and guarding, vomiting, bilateral conjunctivitis, periorbital edema, erythematous fissured lips, erythema and edema in the hands and feet, and a generalized maculopapular rash. Abdominal computerized tomography (CT) scan showed an enlarged pancreas, increased bowel wall thickening, splenomegaly, and mild free fluid. Echocardiogram findings were normal. A repeat COVID-19 PCR test returned positive. Based on clinical course and laboratory results, MIS-C was suspected, leading to treatment with methylprednisolone pulse therapy (30 mg/kg/day for 3 days) followed by prednisolone 1 mg/kg/day for 5 days. The patient's condition improved posttreatment, with complete resolution of symptoms. Short-term (4 weeks) and long-term (6 months) follow-up indicated symptom-free status with improved echocardiography and normal laboratory findings.

### 3.3. Case Three

A 14-year-old girl presented with a chief complaint of fever that began 2 weeks ago, along with abdominal pain, nausea, and vomiting occurring 2 days prior to admission. Due to the presence of free fluid on the abdominal ultrasound report, there was suspicion of a ruptured ovarian cyst, which was further investigated and ruled out. Epigastric pain was noted during the physical examination. She exhibited an oxygen saturation (SpO_2_) level of 88% and ground-glass opacities (GGO) on her chest CT scan and tested positive for COVID-19. She had received remdesivir treatment during a previous admission. Ultimately, due to elevated serum levels of amylase and lipase, she was diagnosed with pancreatitis attributed to COVID-19-associated MIS-C. After 7 days of admission, she was discharged in good health and remained asymptomatic during the 1, 4, and 6-month follow-up assessments.

### 3.4. Case Four

A 3-and-a-half-year-old girl presented with chief complaints of weakness, headache, fever, dyspnea, abdominal pain, and vomiting that began 3 days prior to her visit to the emergency department. Upon physical examination, she exhibited fever, tachypnea, respiratory distress, tachycardia, and an SpO_2_ level of 87% on room air. Significant generalized tenderness, particularly in the right lower quadrant, was noted during the abdominal exam. Diffuse crackles were auscultated in both lung fields. Abdominal ultrasonography suggested appendicitis and right lower quadrant lymphadenopathy. Laboratory data are presented in Table 1, and her blood culture test was negative. Additionally, she presented with hypocalcemia, hyponatremia, and hypomagnesemia. While her chest X-ray was normal, high-resolution computerized tomography (HRCT) revealed bilateral consolidation, collapse in the upper lobes, superior and posterior segments of the lower lobes, and right pleural effusion, leading to treatment with remdesivir. An appendectomy was performed, and the pathological examination confirmed acute appendicitis and periappendicitis without phlegmonous. A positive result for COVID-19 PCR testing at the time of admission was obtained. She was discharged after 4 days, and short-term (4 weeks) and long-term (6 months) follow-ups did not reveal any abnormal laboratory or echocardiographic findings.

### 3.5. Case Five

A 9-year-old girl presented to the emergency department with fever, abdominal pain, weakness, and vomiting that began 5 days prior. Upon examination, she exhibited signs of shock (cool extremities, prolonged capillary filling time, hypotension, and oliguria), tachycardia, tachypnea, periorbital edema, and bilateral nonpurulent conjunctivitis. One month earlier, she had contact with a confirmed case of COVID-19 and experienced flu-like symptoms for 2 days. Abdominal examination revealed generalized tenderness and guarding. Laboratory findings are detailed in Table 1; notably, normal fibrinogen levels, elevated D-dimer, high creatinine, increased lactate dehydrogenase, elevated triglycerides, hypocalcemia, and hypoalbuminemia were observed.

Abdominal ultrasonography indicated moderate to severe free fluid, increased echogenicity of fat, and lymphadenopathy in the right lower quadrant, suggestive of appendicitis. Despite an appendectomy being performed, the pathological examination revealed a normal appendix. Echocardiography showed pericardial effusion, mild tricuspid regurgitation, ectasia of the left anterior descending (LAD) coronary artery, and left main coronary artery (LMCA). Given the suspicion of MIS-C and coronary involvement, she received antibiotics, IVIG, and corticosteroid pulse therapy. She was discharged after a 7-day hospitalization without any clinical complications during her 4-week follow-up. Improvement in ectasia was noted after 2 months; however, mild abnormalities persisted in serial echocardiographic assessments over 6 months. Consequently, she was prescribed acetylsalicylic acid (ASA) as an antithrombotic agent. After 6 months, echocardiography revealed normal coronary arteries, leading to the discontinuation of ASA treatment.

### 3.6. Case Six

A 10-year-old boy presented to the emergency department with fever, headache, weakness, loss of appetite, dyspnea, and cough that had been ongoing for 1 week. During admission, he developed abdominal pain. Upon examination, he exhibited a fever, tachypnea, tachycardia, respiratory distress, and normal auscultation of the heart and lungs. Abdominal examination revealed generalized tenderness, particularly prominent in the right lower quadrant. Abdominal ultrasonography suggested acute or perforated appendicitis. Subsequently, an appendectomy was performed, and the pathological examination reported a normal appendix. Chest X-ray revealed bilateral reticular opacities. His echocardiography results were within normal limits. He was discharged after an 18-day hospitalization, during which he received antibiotics and conservative treatments. Long-term follow-ups at 2, 4, and 6 months showed normal results.

### 3.7. Case Seven

An 8-year-old boy presented with a 4-day history of prolonged fever, followed by nausea, vomiting, and abdominal pain. Upon examination, he displayed a fever, tachypnea, and tachycardia. Abdominopelvic ultrasonography indicated free fluid and reactive lymph nodes in the right lower quadrant interloop. Based on the clinical course and abdominal tenderness, acute abdomen was diagnosed, leading to the performance of a laparotomy. However, the appendix was reported to be normal.

The patient also presented with a cough and decreased SPO_2_ levels to 85%. His mother had tested positive for COVID-19 1 month prior. A chest CT scan revealed bilateral ground-glass opacities and pleural effusion, prompting treatment with remdesivir. Elevated serum levels of creatinine, troponin, and D-dimer were observed. While the COVID-19 RT-PCR test taken at admission reported negative results, his serology for COVID-19 IgG was positive. Echocardiography showed a low ejection fraction. He was diagnosed with MIS-C and received treatment with IVIG (1 g/kg/day for 2 days) and methylprednisolone pulse therapy. After 10 days, he was discharged in good health, and his short-term (4-week) and long-term (4, 4, and 6 months) follow-ups demonstrated normal echocardiography results.

### 3.8. Case Eight

An 11-year-old girl presented to the emergency department with a chief complaint of a 5-day prolonged fever accompanied by subsequent symptoms of headache, nausea, vomiting, skin rash, myalgia, and abdominal pain. Her parents had tested positive for COVID-19 3 weeks prior. Upon examination, she exhibited a fever, abdominal tenderness, rebound tenderness, and signs of meningismus. Abdominal ultrasonography and a CT scan revealed the presence of some free fluid without any indication of appendicitis or other underlying issues. A lumbar puncture examination showed a mild increase in protein levels (50 mg/dL) and a few leukocytes (white blood cell count = 25 cells/mm3, 75% lymphocyte). Empiric antibiotic therapy was initiated until all culture exams returned negative results. Echocardiography revealed mild ectasia in the coronary arteries. The patient was diagnosed with MIS-C and treated with methylprednisolone pulse therapy. She was discharged in good health after 5 days, and her echocardiography results remained normal during follow-up appointments at 2, 4, and 6 months.

## 4. Discussion

In our case series, we have reported eight rare cases of acute abdomen as a presentation of MIS-C. Acute abdomen occurred in children with MIS-C who tested positive for COVID-19 before or during the episode of acute abdomen symptoms. All patients had a fever, GI symptoms, and at least one abnormal finding on abdominal ultrasonography. Cardiac involvement in echocardiography was seen in 50% of cases (Table 2).

COVID-19 is caused by SARS-CoV-2 and is typically characterized by respiratory tract infection symptoms and fever [2]. One of the presentations of COVID-19 in children is MIS-C, which is a hyperinflammatory syndrome with involvement of more than two organs and may present as a Kawasaki-like syndrome [3]. Characteristics of Kawasaki disease and MIS-C have been compared in Table 3, based on the findings of some studies [6, 7, 11, 12, 13, 14]. Although Kawasaki disease and MIS-C have many symptoms in common, there are factors that help discriminate between these conditions. Kawasaki disease occurs in children younger than 5 years, while mucosal and cardiac involvement are common. Yener et al. study's showed that the mean age of MIS-C patients was higher than those with KD while lower than those with sJIA-MAS [9]. In our cases, the majority of them presented Kawasaki-like symptoms, but contrary to typical Kawasaki disease six cases were more than 5 years old.

Some studies suggest that COVID-19 infection in children may result in GI manifestations such as diarrhea, vomiting, and abdominal pain [9, 11, 13, 15]; nevertheless, some MIS-C patients present with acute abdomen [9, 16, 17, 18]. Some characteristics can help physicians to difference between MIS-C and true appendicitis such as recent respiratory infection compatible COVID-19, pericardial effusion, and/or carditis. Moreover, lymphopenia combined with a normal leukocyte count or leukopenia and coagulopathy are commonly found in MIS-C [6, 11, 12, 13, 14]. Laboratory findings in Kawasaki disease usually is leukocytosis.

According to the literature review we conducted, 24 case reports (total cases, 61 patients) on acute abdomen in MIS-C cases are summarized in Table 4 [19, 20, 21, 22, 23, 24, 25, 26, 27, 28, 29, 30, 31, 32, 33, 34, 35, 36, 37, 38, 39, 40, 41, 42]. So, it seems that acute abdomen is not a common symptom in MIS-C. Based on the data from Table 4, all 61 reported cases had fever and GI manifestations such as abdominal pain, vomiting, and diarrhea. The mean age of the total cases was 8.8 years, with 87% of patients being older than 5 years. Seventy-eight percent of cases had C-reactive protein (CRP) levels exceeding 100 mg/dL, and 45% of patients had cardiac involvement (myocarditis or transient coronary involvement). Out of the 61 cases, 47 cases (77%) underwent surgical interventions (laparoscopy, exploratory laparotomy, or appendectomy), but after pathological investigation, appendicitis was confirmed in only 51% of suspected cases, equivalent to 39% of the total cases (Table 4).

In our study, six cases underwent laparotomies, but the rate of true appendicitis in our findings (12%) is significantly lower than what was reported in the literature review of 24 reports (39%) in Table 4. Yock-Corrales [43] et al.'s study revealed that MIS-C can mimic appendicitis symptoms and may present with acute abdomen without any intraoperative findings supporting appendicitis. The pathophysiology of abdominal pain caused by MIS-C could be related to the inflammatory process [44].

The authors' experiences in the present study, along with findings from other studies, suggest that during the COVID-19 pandemic, unexplained abdominal pain and acute abdomen should be considered as an unusual presentation of MIS-C [45, 46]. Data from Table 4 across different studies show that only 39% of all MIS-C cases presenting with acute abdomen had true appendicitis. Therefore, it can be inferred that MIS-C can mimic symptoms of appendicitis. However, it is important to note that the incidence of true appendicitis and/or the co-occurrence of COVID-19 infection and appendicitis may increase in certain subtypes of COVID-19 infections [11].

In our case series, 50% of patients had cardiac involvement, with three cases (37.5%) showing coronary involvement and two cases (25%) displaying evidence of myocarditis. Mild and transient coronary artery involvement and abnormal tapering have been reported in acute infections [47]. Among our cases, three out of four with cardiac involvement exhibited transient coronary ectasia or abnormal tapering during hospitalization. Ultimately, after 4 weeks of follow-up, coronary arteries appeared normal in seven cases. Only one patient (case five) showed evidence of myocarditis and persistent coronary dilation after 6 months. This case presented with relative leukopenia (WBC: 4,400 cells/mm^3^), thrombocytopenia, normal erythrocyte sedimentation rate (ESR), elevated CRP levels, elevated liver enzyme levels, and hyperferritinemia. She manifested symptoms of macrophage activation syndrome (MAS), a severe complication of autoimmune and/or autoinflammatory disorders that can be triggered by viral infections such as Epstein–Barr virus. Furthermore, Kawasaki disease and/or systemic juvenile idiopathic arthritis have similar manifestations to this case [48, 49].

There is a significant relationship between higher virus titer and subsequent strong inflammatory cytokine and chemokine responses, leading to higher mortality and morbidity from COVID-19 [9]. Therefore, controlling the local and systemic inflammatory response to COVID-19 can be just as crucial as antiviral therapy. Experiences from treating SARS and Middle East respiratory syndrome (MERS) have shown that reducing viral load in the early stages and controlling inflammatory responses through immune modulators are effective approaches to improve the prognosis of COVID-19 patients [50].

Our results showed that treatment with corticosteroid pulse therapy alone (three cases), corticosteroid pulse therapy combined with IVIG (three cases), and corticosteroid pulse therapy combined with antibiotic therapy was effective, and all of the patients' symptoms improved. Recent data indicate that using IVIG and antiviral therapy alone not only fails to control hyperinflammation in MIS-C but may also be associated with increased morbidity [5]. According to data from 24 studies presented in Table 4, IVIG and corticosteroids (pulse therapy or anti-inflammatory doses) were administered in 63% and 51% of cases, respectively. Corticosteroids are considered first- or second-line treatments in MIS-C according to recent guidelines [9, 51, 52, 53]. In a study conducted at our center, we found that corticosteroid pulse therapy resulted in low morbidity and zero mortality in a group of severe MIS-C and critically ill children [51]. Similarly, in our current case series, seven out of eight cases treated with methylprednisolone pulse therapy survived without any mortality. This approach was effective as symptoms of acute abdomen resolved even before surgical procedures in two cases. Similar to our experience, in Yener et al.'s study, all patients received corticosteroids, 24.6% as methylprednisolone pulse therapy (10–30 mg/kg, 1–3 days) and 73.4% 2 mg/kg in combination with IVIG [9].

## 5. Conclusion

In patients presenting with acute abdomen, fever, skin rash, and peripheral edema during the coronavirus pandemic, MIS-C should be considered as a differential diagnosis of appendicitis in children with GI manifestations who have a positive or suspicious history of COVID-19 infection. Although acute abdomen is not a common presentation of MIS-C, appendicitis should be considered a high-priority underlying cause in MIS-C patients with GI manifestations. Future studies with long-term follow-up are needed in patients with MIS-C to determine the true incidence of acute abdomen and its subsequent complications. Methylprednisolone and IVIG can rapidly reduce clinical symptoms and shorten the duration of hospitalization without any complications.

## Figures and Tables

**Table 1:** The clinical symptoms and laboratory findings of the study population.

Parameter^*∗*^	Case 1	Case 2	Case 3	Case 4	Case 5	Case 6	Case 7	Case 8
Age/sex	10/F	6/M	14/F	3.5/F	9/F	10/M	8/M	11/F
GI symptoms	+	+	+	+	+	+	+	+
Fever	+	+	+	+	+	+	+	+
Skin involvement	+	+	—	—	—	—	—	+
Mucosal lesion	+	+	—	—	+	—	—	—
Lung involvement	No	No	SpO_2_ = 88%, GGO in chest CT	SpO_2_ = 87%, bilateral consolidation in HRCT	No	Bilateral reticular opacities in chest X-ray	GGO and pleural effusion in Chest CT	No
Echocardiography	NL	Coronary artery ectasia	NL	NL	Pericardial effusion, coronary artery dilation	NL	Myocarditis and low ejection fraction	Coronary artery ectasia
WBC/lymph counts (cells/mm^3^)	11,700 ↑/1,094 ↓	6,050/1,200	5,920/1,680	12,600 ↑/1,120↓	4,400/580↓	7,280/390↓	25,070 ↑/3,500↓	14,050 ↑/950↓
Hemoglobin (g/dL)	9.4	10.6	10.3	9.9	8.9	9.3	9.9	10.2
Platelet count (cells/mm^3^)	100,000 ↓	141,000 ↓	181,000	210,000	61,000 ↓	134,000 ↓	243,000	100,000 ↓
ESR (mm/hr)	31 ↑	24 ↑	9	54 ↑	15	62 ↑	74 ↑	90 ↑
CRP (mg/dL)	134 ↑↑	28 ↑	37 ↑	270 ↑↑	125 ↑	196 ↑↑	24 ↑	81 ↑
AST (U/L)	12	32	138↑	28	110 ↑	86 ↑	54 ↑	59 ↑
ALT (U/L)	11	18	61 ↑	32	86 ↑	67 ↑	50 ↑	29
Albumin (g/dL)	2 ↓	2.8 ↓	3.5	3.7	2.7 ↓	3.5	3.1 ↓	3.3 ↓
Amylase (U/L)	46	94	270 ↑	56	52	42	144 ↑	55
Lipase (U/L)	104 ↑	208 ↑	1,168 ↑↑	76 ↑	67 ↑	58	72 ↑	50
Ferritin (g/mL)	—	720 ↑	480 ↑	760 ↑	1,350 ↑↑	950 ↑	890 ↑	699 ↑
COVID-19 evidence	PCR+, IgG +, IgM−	PCR +	PCR +	PCR +	Contact with a Pos. case ^*∗∗*^	PCR +	Contact with a Pos. case, IgG +	Parents history +
Abdominal sonography								
Free fluid	+	+	—	—	+	+	+	+
Bowel wall thickening	+	+	+	—	—	Acute appendicitis	—	—
Lymphadenopathy	+	—	—	+	+	—	+	+
Surgery/pathologic result	Laparotomy/(bowel wall thickening)	—	—	Appendectomy/acute appendicitis	Appendectomy/no appendicitis	Appendectomy/no appendicitis	Laparotomy/no appendicitis	Laparotomy/no appendicitis
Specific treatment	Corticosteroid pulse therapy	IVIG + corticosteroid pulse therapy	Corticosteroid pulse therapy	Corticosteroid pulse therapy	IVIG + corticosteroid pulse therapy	—	Antiviral therapy + IVIG + corticosteroid pulse therapy	Antibiotic therapy + corticosteroid pulse therapy
Follow-up (6 months after discharge)	Mild persistent thrombocytopenia	NL	NL	NL	Mild abnormal deficit for 6 months	NL	NL	NL

Abbreviations. ALT, alanine transaminase; AST, aspartate transaminase; CRP, C-reactive protein; CT, computerized tomography; ESR, erythrocyte sedimentation rate; GGO, ground-glass opacities; GI, gastrointestinal; HRCT, high-resolution computerized tomography; IgG, immunoglobulin G; IgM, immunoglobulin M; IVIG, intravenous immunoglobulin; lymph, lymphocyte; NL, normal; PCR, polymerase chain reaction; SpO_2_, oxygen saturation of blood; WBC, white blood cell.  ^*∗*^Laboratory tests reference normal ranges: WBC, 4–10 ×1,000 cells/uL; lymph, 20%–40% of WBC count; hemoglobin, 11–16 g/dL; platelets, 150–450 × 1,000 cells/mm^3^; ESR, 0–10 mm/hr; CRP, <6 mg/dL; AST, 10–31 U/L; ALT, 10–31 U/L; albumin, 3.5–5.2 g/dL; amylase, <100 U/L; lipase, 13–60 U/L; ferritin, 30–220 g/mL.  ^*∗∗*^Contact with a Pos. case: The patient recently encountered with a newly verified COVID-19 positive patient. ↑: mild increase, ↑↑: significant increase, ↓: decrease.

**Table 2:** The frequency of clinical and paraclinical findings in our eight patients.

Parameter	Frequency
Sex (male/female)	3/5
Gastrointestinal symptoms	100%
Fever	100%
Skin involvement	37.5%
Mucosal involvement	37.5%
Abdominal ultrasonography findings	100%
(i) Free fluid (ii) Bowel wall thickening (iii) Lymphadenopathy	75% 37.5% 62.5%
Abnormal echocardiography	50%
Leukocytosis (>10,000 cells/mm^3^)	50%
Leukopenia	0%
Lymphopenia (<1,500 cells/mm^3^)	75%
Anemia (Hgb < 12 g/dL)	100%
Platelets < 150,000 cells/mm^3^	63.5%
ESR > 20 mm (hr)	75%
Elevated CRP	100%
CRP > 100 mg/dL	50%
Elevated AST	62.5%
Elevated ALT	50%
Serum albumin < 3.5 g/dL	87.5%
Elevated amylase > 110 U/L	25%
Elevated lipase > 140 U/L	25%
Elevated serum ferritin > 350 g/mL	100%
Positive COVID-19 evidence	100%
True appendicitis	12.5%

Abbreviations. ALT, alanine transaminase; AST, aspartate transaminase; CRP, C-reactive protein; ESR, erythrocyte sedimentation rate.

**Table 3:** Comparison of characteristics of Kawasaki disease and multisystem inflammatory syndrome in children (MIS-c).

Parameter	Kawasaki disease	MIS-c	COVID-19 infection
Mean age of patients	Less than 5 years	More than 5 years	Any age
Skin involvement	Common	Common	Very rare
Mucosal involvement	Common	Uncommon	Very rare
Gastrointestinal involvement	Uncommon	Common	Related to subtype
Coronary artery involvement	Common	Uncommon	Very rare
Pericardial effusion/carditis	Rare	Common	Rare
Central nervous system involvment	Uncommon	Common	Very rare
Shock and/or ICU admission	Uncommon	Very common	Uncommon
WBC count	Leukocytosis	Normal or leukopenia	Normal or mild increase
Lymphocyte count	Normal	Lymphopenia	Lymphopenia
Platelet count	Normal or increased	Lower limit of normal or thrombocytopenia	Normal or mild thrombocytopenia
CRP (mg/L)	>50	>100	<50
Serum ferritin (*µ*g/L)	Mild increased (250–400)	Hyperferritinemia (moderate to severe) (>500)	Normal (<250) or mild increased
Liver transaminase	Mild increase	Moderate to severe increase	Normal
Coagulopathy (elevated D-dimer, prolong PT)	Rare	Common	Uncommon
Response to IVIG	Common and dramatically	Relative	No indication
Response to methylprednisolone pulse therapy	Common and dramatically	Common and dramatically	No indication

Abbreviations. ICU, intensive care unit; CRP, C-reactive protein; IVIG, intravenous immunoglobulin; MIS-c, multisystem inflammatory syndrome in children; PT, prothrombin time; WBC, white blood cell. *Note*. Table information is summarized according to some reference studies [6, 7, 11, 12, 13, 14].

**Table 4:** Review of demographics and clinical and paraclinical findings of MIS-c patients with acute abdomen symptoms in different studies^*∗*^.

Study (reference)	No. of patients	Mean age (year)	Age >5 year	Male/female	WBC > 150,000	CRP > 100 mg/L	Ferritin > 500	Ultrasound or CT scan finding				Cardiac involvement	Treatment		Surgery	True appendicitis
								Free fluid	Bowel wall thickening	Lymphadenitis	Acute appendicitis		IVIG	Steroid		
Our study (%)	8 (100%)	8.5	7 (87%)	3/5	1 (12%)	4 (50%)	6 (75%)	6 (75%)	3 (38%)	5 (62%)	1 (12%)	4 (50%)	3 (38%)	7 (87%)	6 (75%)	1 (12%)
Tullie et al. [19]	8	10.1	7	5/3	3	7	—	2	5	8	0	1	4	4	0	0
Lopez et al. [20]	8	9.5	7	3/5	—	8	0	0	1	0	1	5	8	2	8	3
Hofto et al. [21]	4	12	3	2/2	2	4	2	1	0	0	2	1	4	2	2	2
Garcia et al. [22]	3	5.3	1	2/1	1	3	0	2	1	1	2	0	2	1	3	3
Nurnaningsihn et al. [23]	6	8.3	5	4/2	6	4	—	—	0	0	4	—	0	0	6	6
Manz et al. [24]	3	8	3	2/1	0	3	1	2	2	3	1	0	2	3	3	2
Alotaibi et al. [25]	2	8.5	2	1/1	1	0	2	1	0	0	2	0	2	2	2	1
Maskari et al. [26]	2	5.5	2	2/0	0	1	1	0	0	1	0	0	2	2	1	0
Anderson et al. [27]	2	8.5	2	0/2	1	2	1	1	0	1	0	2	2	0	2	1
Anderson et al. [28]	1	11	1	M	0	0	—	1	0	1	0	1	1	1	1	0
Aslan et al. [29]	1	12	1	F	—	—	—	0	1	1	0	—	1	0	0	0
Salaheldin et al. [30]	1	12	1	M	0	1	1	—	—	—	—	1	1	1	1	0
Falqueto et al. [31]	1	12	1	M	0	0	—	1	1	1	—	1	1	0	1	1
Nurullayev et al. [32]	1	13	1	M	0	1	—	0	0	0	1	—	0	0	1	1
Ahsanuddin et al. [33]	1	7	1	F	1	0	—	1	0	0	0	—	—	—	1	1
Panko et al. [34]	1	6	1	F	1	1	—	—	—	—	—	—	0	0	1	0
Serra et al. [35]	1	9	1	M	1	1	0	0	0	0	1	0	0	0	1	1
Uğur et al. [36]	1	5	1	M	1	1	0	0	1	0	1	1	1	1	1	0
Trevisan et al. [37]	1	15	1	M	0	1	—	1	1	1	0	1	1	1	1	0
Martin et al. [38]	1	4	0	F	0	1	0	1	0	0	1	1	1	1	1	0
Rinaldi et al. [39]	1	10	1	M	0	1	0	—	1	0	0	1	1	0	1	0
Hwang et al. [40]	1	16	1	F	0	1	—	0	0	0	1	1	1	1	1	0
Jackson et al. [41]	1	9	1	F	0	1	1	1	0	0	1	1	1	1	1	0
Kareva et al. [42]	1	11	1	M	1	1	0	1	0	0	1	1	0	1	1	1
Total (%)	61 (100%)	8.8	53/61 (87%)	33/28	20/52 (38%)	47/60 (78%)	11/39 (38%)	22/52 (42%)	17/59 (37%)	23/59 (44%)	20/58 (34.5%)	23/51 (45%)	39/60 (65%)	31/60 (52%)	47/61 (77%)	24/61 (39%)

Abbreviations. CRP, C-reactive protein; F, female; IVIG, intravenous immunoglobulin; M, male; MIS-c, multisystem inflammatory syndrome in children; WBC, white blood cells.  ^*∗*^Data are shown as number of patients, except for mean age (year). *Note*. This table data are a summary of our literature review, and all the references were recruited from PubMed, Google Scholar, and Embase.

## Data Availability

Requests for access to the data that support this report should be made to the corresponding author, Vahid Ziaee.
